# Molecular Targeted Agents for Gastric Cancer: A Step Forward Towards Personalized Therapy

**DOI:** 10.3390/cancers5010064

**Published:** 2013-01-21

**Authors:** Esther Una Cidon, Sara G Ellis, Yasir Inam, Sola Adeleke, Sara Zarif, Tom Geldart

**Affiliations:** Medical Oncology Department, The Royal Bournemouth and Christchurch Hospitals NHS Foundation Trust, Castle Lane East, BH7 7DW Bournemouth, Dorset, UK; E-Mails: saragellis@gmail.com (S.G.E.); drinam3@gmail.com (Y.I.); sola_adeleke.2003@yahoo.com (S.A.); saramohmand@gmail.com (S.Z.); Tom.Geldart@rbch.nhs.uk (T.G.)

**Keywords:** gastric cancer, molecular targeted agents, monoclonal antibody, tyrosine kinase inhibitor

## Abstract

Gastric cancer (GC) represents a major cancer burden worldwide, and remains the second leading cause of cancer-related death. Due to its insidious nature, presentation is usually late and often carries a poor prognosis. Despite having improved treatment modalities over the last decade, for most patients only modest improvements have been seen in overall survival. Recent progress in understanding the molecular biology of GC and its signaling pathways, offers the hope of clinically significant promising advances for selected groups of patients. Patients with Her-2 overexpression or amplification have experienced benefit from the integration of monoclonal antibodies such as trastuzumab to the standard chemotherapy. Additionally, drugs targeting angiogenesis (bevacizumab, sorafenib, sunitinib) are under investigation and other targeted agents such as mTOR inhibitors, anti c-MET, polo-like kinase 1 inhibitors are in preclinical or early clinical development. Patient selection and the development of reliable biomarkers to accurately select patients most likely to benefit from these tailored therapies is now key. Future trials should focus on these advances to optimize the treatment for GC patients. This article will review recent progress and current status of targeted agents in GC.

## 1. Introduction

Gastric cancer (GC) is an aggressive disease and represents a major cancer burden worldwide. Though the absolute incidence of GC has declined globally, it is still the fourth most prevalent malignancy and the second leading cause of cancer-related death around the World [1].

Surgery is the only potentially curative treatment, but even after radical excision most patients will ultimately recur in regional or distant sites with a 5-year survival rate of only 20–25% [2,3]. Although surgery is appropriate for patients with resectable disease, most GC patients present with locally advanced or metastatic disease which is not amenable for radical surgery [4]. Multimodality treatment with neoadjuvant or adjuvant regional and systemic therapies is now a standard of care but despite the integration of combined therapies, overall survival remains low with 5-year survival rates of 30–36% [5,6].

For patients presenting with metastatic disease, chemotherapy remains the mainstay of palliative treatment with median survival figures of between 9 and 14 months [6,7].

The development of new treatment techniques continues to be a key priority. Recent progress in the understanding of molecular pathways involved in a variety of cancers has led to the discovery of new targeted therapies. Trastuzumab has already been approved as standard care for HER2-positive GC patients, according to the results of the Trastuzumab for Gastric Cancer (ToGA) clinical trial, where it demonstrated its therapeutic utility [8]. Further candidate molecules related to cell growth, invasion, apoptosis, angiogenesis and metastasis are emerging as a new hope in the treatment strategy for many common malignancies such as breast, colorectal, lung, renal and melanoma. Agents targeting molecules such as the epidermal growth factor receptor (EGFR), vascular endothelial growth factor (VEGF) receptor, P13k/Akt/mTor pathway, insulin-like growth factor receptor (IGFR), c-Met pathways, fibroblast growth factor receptor (FGFR) and other pathways are promising candidates for targeted therapy for GC and are now in clinical development.

Thus, a new era in the treatment of GC and GEJ cancer has been opened up. Historically, most phase III clinical trials have been performed without patient selection. Predictive biomarkers and personalisation of therapy is now of utmost importance and translational research now needs to be at the heart of clinical trial design.

This review will introduce the recent investigations and current status of targeted agents for the treatment of advanced GC and GEJ cancers (Table 1).

**Table 1:** Molecular targeted agents for gastric cancer and their target.

Target	Drugs
**Anti-EGFR**	
***EGFR antibodies***	
	Cetuximab
	Panitumumab
	Nimotuzumab
	Matuzumab
***EGFR TKI***	
	Erlotinib
	Gefitinib
**HER-2 inhibitors**	
***HER-2 humanized monoclonal antibody***	
	Trastuzumab
***HER-2 TKI***	
	Lapatinib
**Antiangiogenic therapy**	
***Anti-VEGF monoclonal antibody***	
	Bevacizumab
	Ramucirumab
***VEGFR TKI***	
	Sorafenib
	Sunitinib
	Cediranib
	Apatinib
	Telatinib
**Other targeted agents**	
***PI3k-Akt-mTOR-targeted therapy***	
	Everolimus
***Polo-like kinase 1 inhibitors***	
***HGF-c-Met Pathway and FGFR Pathway***	
	Tivantinib
	Rilotumumab
***Fibroblast growth factor receptor (FGFR)***	
	Dovitinib

## 2. Epidermal Growth Factor Receptor (EGFR)

The epidermal growth factor (EGF) is a protein which stimulates cell growth, proliferation, and differentiation by binding to its receptor EGFR [9].

The EGFR is the cell-surface receptor for members of the EGF family [10]. When ligands bind to the extracellular domain, this interaction leads to the EGFR activation which in turn homodimerizes, resulting in phosphorylation of the intracellular tyrosine kinase (TK). This eventually results in a series of intracellular signals cascades, including the central Ras/Raf/mitogen activated protein kinase (MAPK) or the Akt/mTOR pathway [11]. All these cascades are potent regulators of intracellular/intercellular processes, such as cell cycle progression, apoptosis, proliferation, angiogenesis and metastasis. These activities have constituted the rationale for the development of agents able to block the EGFR activity [12]. EGFR is highly expressed and activated in many cancer types. In GCs, EGFR overexpression by immunohistochemistry (IHC) or gene amplification by fluorescent *in situ* hybridization (FISH) occurs in 50–63% of patients [13] and is known to be associated with increased invasion, a poorly differentiated histology and shorter survival [14,15,16,17,18].

The EGFR inhibitors that have been tested in clinical trials are monoclonal antibodies such as cetuximab, panitumumab, nimotuzumab and matuzumab, and tyrosine kinase inhibitors (TKI) including gefitinib and erlotinib.

### 2.1. EGFR Inhibitors: Monoclonal Antibodies

#### 2.1.1. Cetuximab

Cetuximab is a recombinant, human/chimeric IgG1 monoclonal antibody (mAb) directed against the EGFR [19]. It binds to the extracellular domain of EGFR in its inactive configuration and competitively inhibits its binding to other ligands by blocking the binding region. This mAb-receptor union prevents receptor dimerization and therefore blocks ligand-induced EGFR TK activation. Cetuximab also induces EGFR internalization, downregulation, and degradation.

Cetuximab has also been shown to mediate Ab dependent cell cytotoxicity (ADCC) which may also contribute to its anticancer activity. This antibody has been evaluated in many phase II studies in patients with advanced GC and GEJ cancer either as monotherapy or combined with chemotherapy.

##### 2.1.1.1. First-Line Setting

In first-line setting Cetuximab has been evaluated in combination with different regimens of chemotherapy, such as 5FU, folinic acid, irinotecan (iri) (FOLFIRI) [20], docetaxel/cisplatin (cis) [21], FOLFOX [22], (capecitabine (cape), oxaliplatin (ox) (XELOX) [23], weekly iri, infusional 5FU, leucovorin (FUFIRI) [24], continuous infusion high dose 5FU/leucovorin /cis [25], cape/cis [26], and ox/iri [27].

With these combinations, tumor response rates (RR) ranged between 41% and 69% and median time to progression (TTP) ranged between 5 and 8.5 months with a median overall survival (OS) between 9 and 16.6 months. Overall, serious cetuximab-related side-effects observed were skin rash, diarrhea, and infusion reactions, all manageable, but there was also one treatment-related death in the FOLCETUX trial [20].

The relationship between RR and EGFR expression has not been well established, and there have been reported contradictory results [28,29].

Pinto *et al*. (DOCETUX trial) reported the results of a combination with cis and docetaxel for advanced GC and GEJ cancers in a non-selected population. The disease control rate (DCR) was f 76.5%, median TTP of 5 months (95% confidence interval (CI): 3.7–5.4%) and median OS of 9 months (95% CI 7–11). More interestingly was the fact that in the setting of stable disease after 6 cycles, those treated with maintenance cetuximab compared with no-maintenance showed a trend, although non-significant, for longer TTP of 9.2 *vs.* 6.6 (*p* = 0.10), and OS of 19.8 *vs.* 7.7 (*p* = 0.22).

The FOLCETUX trial, carried out in a EGFR positive population, showed a median TTP of 8 months and OS of 16 months respectively [20].

Generally both trials showed good tolerance, being neutropenia the most frequent grade 3/4 toxicity, with one toxic death in the DOCETUX trial due to neutropenia sepsis [21] (See Table 2).

**Table 2:** Phases II and III of cetuximab combined with chemotherapy for advanced GC in first-line setting.

Author/Ref.	Type of study	N	Selected by EGFR positive	Type of chemo	% RR	TTP months	OS months
Pinto *et al*. [20]	Phase II	38	Yes, by Inmunohistochemistry (IHC)	FOLFIRI	44.1	8	16
Pinto *et al*. [21]	Phase II	72	Not required	Cis + docetaxel	41.2	5	9
Han *et al*. [22]	Phase II	38	Not required	FOLFOX-6	50	5.5	9.9
Enzinger *et al*. [30]	Phase II	245	Not required	ECF	58	5.6	10
				IC	38	5	8.6
				FOLFOX	51	5.7	10
Kim *et al*. [29]	Phase II	44	Not required	XELOX	52.3	6.5	11.8
Kanzler *et al*. [24]	Phase II	49	Not required	FUFIRI	42	8.5 ^a^	16.6
Yeh *et al*. [25]	Phase II	35	Not required	Cis + 5FU + leuco-vorin	68.6	11 ^a^	14.5
Wöll *et al*. [27]	Phase II	51	Not required	IROX	63	6.2	9.5
Zhang *et al*. [26]	Phase II	54	Not required	Cis + cape	48.1	5.23	
Merck-Serono [31]	Phase III	904	Not required	Cis + cape + cetuximab/placebo	29/30	4.4/5.6 ^a^	9.4/10.7

^a^ PFS.

More recently the CALGB80403/ECOG 1206 randomized phase II trial has compared the tumor RR in patients receiving cetuximab combined with three different chemotherapy regimens [epirubicin, cis, fluorouracil (ECF), *vs.* iri plus cis (IC) *vs.* FOLFOX], all in non-selected patients. Preliminary reports showed RR of 57.8, 45.6, and 53.6%, respectively, and OS of 11.5, 8.9, and 12.4 months, respectively. Moreover, cetuximab combined with FOLFOX had one of the most favorable safety profiles [30].

Based on these promising results a phase III clinical trial was initiated. The open-label, international, randomized, controlled, multicenter EXPAND trial investigated the combination of cape and cis as a first line treatment in advanced GC and GEJ cancer with or without cetuximab. This study included 904 patients with unresectable advanced GC or GEJ cancer who had not received any prior treatment with chemotherapy or radiotherapy. The study unfortunately did not meet the primary end-point of an improvement in PFS (4.4 *vs*. 5.6 months), neither increased the OS (9.4 *vs*. 10.7 months), with no significant differences in RR (29 *vs.* 30%) with and without cetuximab respectively [31]. Once again the population was non-selected according to any specific biomarker which could have influenced the results.

Interesting is the fact that due to its favorable safety profile, cetuximab has been also evaluated in a combination with carboplatin and paclitaxel with concurrent radiation for radical treatment of GEJ cancers. Although the population in this study was non-selected by EGFR status, authors reported high rates of complete remission (70%) though data on OS were not included. More interesting was even the finding that there was no an increase in esophagitis or other radiation-enhanced toxicity [32].

##### 2.1.1.2. Second-Line Setting

When used in second or advanced line setting, cetuximab as monotherapy appears to have minimal activity in metastatic GC and GEJ cancers although again tumor expression of EGFR was not required to participate in these trials. In this scenary cetuximab monotherapy has been shown minimal RR and an OS ranging from 3.1 to 4 months [33,34].

For docetaxel-refractory patients, when cetuximab was added to docetaxel, the RR was only 6% and the median PFS was short (2.1 months) [35].

The combination of cetuximab and iri in heavily pre-treated patients achieved an overall RR of 23% [36]. However, despite this interesting finding, the contribution of cetuximab in this combination is unclear because of the documented activity of single agent iri in these patients [37]. In fact, Thuss-Patience *et al*. carried out a prospective multicenter randomized phase III trial aiming to compare iri alone in a second line advanced GC setting with best supportive care (BSC) and the results showed a significant benefit in OS for iri, 123 *vs.* 72.5 days (HR = 2.85 (95% CI 1.41–5.79), Log rank test (two-sided): *p* = 0.0027) [38].

To date, in an unselected population of patients with GC/GEJ cancer, the contribution of cetuximab does not appear to be substantial. This means that additional retrospective/prospective translational studies may in time confirm whether a subpopulation of patients derive specific benefit from the addition of this antibody.

#### 2.1.2. Panitumumab

Panitumumab (P) is a fully human IgG2 mAb targeting the EGFR. In GC and GEJ cancer the EGFR gene copy number, as assessed by FISH might be a predictive biomarker of clinical activity with this agent [39].

The REAL-3 phase III randomized trial evaluated the addition of P to a standard regimen of epirubicin, ox, and cape (EOC) in an unselected population with GEJ cancer [40,41,42]. Disappointingly, the addition of P to EOC chemotherapy was associated with worsening of OS (8.8 months compared with 11.3 months for the standard EOC regimen (HR = 1.37; *p* = 0.013). These outcomes represent a meaningful 37% increase in the risk of death in the P arm. There was also a trend toward shorter PFS with P (6.0 *vs*. 7.4 months; HR = 1.22; *p* = 0.068). Authors concluded that these findings may be in part due to lowered doses of Ox and Cape in the modified EOC+P regimen and therefore further studies would be necessary [40,41,42].

Whilst there was no significant difference in the overall incidence of grade 3 or higher adverse events between the two arms, the arm containing P was associated with an increased rate of grades 3 and 4 diarrhoea, skin rash and mucositis but reduced rates of neutropenia and peripheral neuropathy.

Once again, additional retrospective/prospective translational studies may shed light on whether certain subpopulations of patients could derive specific benefit from the addition of this antibody.

#### 2.1.3. Nimotuzumab

Nimotuzumab (N) is a humanized IgG1 anti-EGFR mAb which has demonstrated efficacy in absence of severe skin toxicity caused by other EGFR-binding therapies. A phase II open-label, multicenter, randomized study compared N plus iri *vs.* iri alone in patients with advanced or metastatic GC refractory to 5FU based regimen. The primary end-point was PFS. The preliminary results presented in ASCO annual meeting 2011 did not show a clear benefit with the addition of N but suggested a trend towards a potential benefit in EGFR positive patients [43].

In 2012 another randomized phase II clinical trial with the combination of cis and S-1with/without N in first-line GC patients was presented in ASCO annual meeting. 62 patients were included and the ORR was 50 *vs.* 63% with and without the antibody respectively. Median TTP was in favour of the combination with N (5 *vs.* 3 months). Once again, further studies are warranted to draw final conclusions [44].

#### 2.1.4. Matuzumab

Matuzumab (M) is a humanized IgG1 mAb against the EGFR. Rao *et al.* carriet out a phase I study of M combined with the ECX regimen (epirubicin/cis/cape) as first-line therapy for patients with EGFR positive GC and GEJ cancer by IHC1atients screened, 47% had EGFR-positive tumors. The objective RR was 65%, and the median TTP was 5.2 months. The treatment was well tolerated, with fatigue being the major dose-limiting toxicity [45]. Of note, the TTP in this study was inferior to the PFS obtained in a phase III clinical trial of ECX without a targeted agent (6.2 months), reported by the same group [46].

An European randomized phase II trial (Matrix EG) carried out in GC and GEJ cancer with EGFR overexpression by IHC has evaluated ECX with or without M (Table 3). Preliminary results showed a RR of 58% *vs.* 31%, TTP of 7.1 *vs.* 4.8 months, and OS of 12.2 *vs.* 9.4 months, in favour of M [47].

**Table 3:** Clinical Trials with anti-EGFR agents (non-cetuximab) in advanced GC and GEJ cancers.

Author	Type of study	N	Selected by EGFR	Line of treatment	Treatment	% RR	TTP months	OS months
Okines *et al*. 2010 REAL-III [42]	Phase II–III	200	Not required	First-line	EOC + P	_	7.4	11.3
					EOC		6	8.8
Kim *et al*. 2011 [43]	Phase II	82	Not required	Advanced-line	Iri + N	_	2.4 ^a^	9.7
					Iri		2.8 ^a^	7.5
Wang *et al.* [44]	Phase II	62	Not required	First-line	Cis + S-1 + N	50	5	_
					Cis + S-1	63	3	_
Rao *et al*. MATRIX [47]	Phase II	35	Positive by IHC	First-line	ECX + M	58	7.1	12.2
					ECX	31	4.8	9.4

^a^ PFS.

### 2.2. EGFR Tyrosine Kinase Inhibitors (TKI)

Erlotinib and gefitinib are two small oral molecules of TKIs class that inhibit EGFR autophosphorylation and signal transduction [48].

#### 2.2.1. Gefitinib

Gefitinib (ZD1839) is an orally active EGFR. In a phase II study of gefitinib monotherapy (at 250 mg/day or 500 mg/day), 75 unselected patients with previously treated GC and GEJ cancers were treated, and the rate of disease control (DCR) was only 18.3%. This study was designed to assess “*in situ*” the biologic activity of the EGFR TKI, and gefitinib reached enough tumor concentrations to inhibit EGFR activation, however, this was not translated into clinical benefit [49]. Another phase II evaluating Gefitinib used in combination with cis+fluorouracil and radiotherapy in patients with locally advanced esophageal and GEJ cancer, as neoadjuvant treatment did not increase pathological complete RR, but 3-year OS increased compared with historical controls showed a benefit (42% *vs*. 28%) [50].

#### 2.2.2. Erlotinib

In a phase II trial (SWOG 0127) of erlotinib used as a first line treatment in 70 unselected patients with advanced GC and GEJ cancer, the RR was 9% in GEJ cancer but no responses were seen in patients with GC. The OS was 6.7 and 3.5 months in patients with advanced GEJ cancer and GC. Authors concluded that erlotinib was active in patients with GEJ cancer, but apparently inactive against GC [51,52]. Moreover, when EGFR status was evaluated, it appeared not to be predictive of the outcome.

It has been suggested that the lack of EGFR TKIs inhibitors activity in GC may be related to the different etiology seen by locations. In fact GEJ cancers are associated with Barrett’s esophagus, while GC with *Helicobacter pylori* infection. Moreover, the different molecular pathways targeted by these agents could be differentially expressed in proximal *vs.* distal cancers [53].

## 3. Human Epidermal Growth Factor Type 2 (HER-2) Targeting Agents

HER2 is a member of the EGFR family. When ligands bind to the extracellular domains of these receptors, this interaction leads to homodimerization and heterodimerization of the EGFRs, followed by tyrosine autophosphorylation. In this cascade HER2 plays a coordinating role since each receptor with a specific ligand prefers HER2 as its heterodimeric partner.

Overexpression of HER2 is seen in many types of human tumors including GC and GEJ cancers. Overexpression of HER2-neu evaluated by IHC and FISH in GC and GEJ cancers has been reported in 7.5–22.9% of tumors. The largest data set of 3,883 advanced GC samples found Her-2 positivity, measured by inmunohistrochemistry and/or FISH, in 22.9% of samples. Her-2 positivity was higher in GEJ cancers than GC (33.2 *vs*. 20.9%, *p* = 0.001), and higher in intestinal than in diffuse/mixed cancer (32.2 *vs*. 6.1%/20.4%, *p* = 0.001) [9,54,55,56,57,58].

HER2 targeting agents include the monoclonal antibody trastuzumab (T) and the tyrosine kinase inhibitor lapatinib (L).

### 3.1. Anti-Her-2/neu mAb: Trastuzumab

T is a humanized monoclonal antibody against HER2. In preclinical studies, T inhibits the growth of HER2-positive GC cell lines and this effect is enhanced when combined with cytotoxic agents active in human GC such as cis, cape, iri, doxorubicin and taxanes [59,60,61].

The preliminary results of a small phase II study with T and cis in 21 advanced GC patients with Her-2 overexpression demonstrated RR of 35%, with disease control rate (DCR) of 52% [62].

Safran *et al.* carried out another small phase I/II trial in which increasing doses of T were combined with cis/paclitaxel and radiation in patients with GEJ cancer HER2 overexpression of 2+ or 3+ by IHC. Nineteen patients were enrolled. 74% of them had either 3+ of HER2 or an increased HER2 gene copy number by FISH. Among them a 57% had a clinical complete response. Six of these patients underwent surgery, and 3 were found to have a pathological complete response. The median OS for all patients was 24 months and the 2-year survival was 50%. There was no cardiac toxicity in this trial [63].

The ToGA study was the first phase III, randomized controlled trial to evaluate T efficacy and safety in Her-2-positive advanced GC and GEJ cancers. The study was designed to investigate if adding T to a combination of cis and 5FU or cape could improve OS. Tumors from 3807 patients were evaluated for Her-2 positivity. 22.1% were positive, and 594 patients were randomized. At randomization, patients were stratified according to Eastern Cooperative Oncology Group performance status, chemotherapy regimen, extent of disease, primary cancer site, and measurability of disease. Median follow-up was 18.6 months in the T plus chemotherapy arm and 17.1 in the chemotherapy alone group. There was a statistically significant difference in RR in favour of T (47.3% in the trastuzumab plus chemotherapy group compared with 34.5% in the chemotherapy arm (*p* = 0.0017) Median PFS and OS favored also to the T containing arm (PFS 6.7 *vs.* 5.5 months, HR 0.71, 95% CI 0.59–0.85; *p* = 0.0002; OS 13.8 *vs.* 11.1 months respectively; *p* = 0.0046, HR = 0.74, 95% CI 0.60–0.91) [8].

An exploratory, post-hoc analysis showed that T plus chemotherapy substantially improved OS (16 months) in patients with high expression of HER2 protein (IHC 2+ and FISH positive or IHC 3+) compared with patients with low expression (IHC 0 or 1+ and FISH negative). No unexpected side-effects were seen in the T plus chemotherapy arm. Although an increase in incidence of asymptomatic left ventricular ejection fraction decrease (5% *vs*. 1%) was noted, no clinical cardiac failure was seen.

The ToGA trial represents a landmark phase III trial of a targeted therapy in GC and GEJ cancer and is the first such study to show significant improvent in OS for a preselected patient population. T combined with chemotherapy is therefore a new standard option for the treatment of HER2-positive advanced GC and GEJ cancer [8] (See Table 4).

### 3.2. Anti-HER-2 TKI: Lapatinib

L is a dual tyrosine kinase inhibitor of EGFR and HER2. In GC and GEJ cancers, L may provide an effective molecularly targeted therapy in patients with T-resistant tumors. Several phase II studies have evaluated the efficacy of L monotherapy in GC. The SWOG-S0413 investigated L in 47 chemotherapy naive, unselected patients, showing modest activity. Only 7% of patients had a PR, and 20% had SD. TTP was 2 months and OS 5 months [64]. Another one carried out in a variety of malignancies with Her-2 amplification, considered to be refractory to standard therapy, assigned 16 patients with GEJ to L therapy, using a randomized discontinuation strategy for those who had SD. One patient had a durable complete response which lasted at least for 15 months and another patient had SD maintained at week 36 [65].

Hecht *et al*. evaluated this treatment in 21 pretreated patients, but no objective response was seen though two patients experienced SD for 5 and 9 months [66].

Other studies were performed with L in combination with different regimens of chemotherapy. Lenz *et al*. combined L with cape in first-line. The patients were unselected and the results showed a RR of 24% in 58 advanced GC patients [67].

A number of studies are currently evaluating L in combination with chemotherapy in advanced GC. EORTC-40071 is a randomized, placebo-controlled phase II study evaluating L in combination with ECF or ECX. This study will prospectively explore the roles of Her-2 and EGFR status [68]. L is also being tested in TYTAN, an open-label, randomized phase III study comparing paclitaxel with and without L in advanced GC patients expressing Her-2 as second-line therapy [69]. Another phase III study (LOGiC; L Optimization Study in HER2 Positive Gastric Cancer; LOGIC) is comparing cape and ox with/without L in a first-line setting [70] (See Table 4).

**Table 4:** Clinical trials with anti-HER-2 agents: T and L.

Author	Type of study	N	Line of treatment	Treatment	% RR	TTP months	OS months
Bang *et al*. [8]	Phase III	594	First-line	Cisp + 5FU/capecit+ T	47.3	6.7	13.8
	ToGA		(selected)	Cis + 5FU/cape	34.5	5.5	11.1
Iqbal *et al.* [64]	Phase II	47	First-line	L	7	2	5
			(unselected)				
Galsky *et al*. [65]		16	Advanced-line	L	6	_	_
Lenz *et al*. [67]	Phase II	58	First-line	L + cape	24	_	_
			(unselected)				
TYTAN [69]	Phase III	_	Second-line	Paclitaxel + L	_	_	_
			(Selected)	Paclitaxel			
LOGIC [70]	Phase III	_	First-line	Capecit + oxali + L	_	_	_
			(Selected)	Capecit + oxali			

## 4. Vascular Endothelial Growth Factor (VEGF)

Tumor growth and metastasis has been strongly linked with angiogenesis in most human tumors and the vascular endothelial growth factor (VEGF) is the most potent and specific angiogenic factor identified [71].

There are several members included in the VEGF family, namely VEGF-A, B, C, D, E and placenta growth factor (PGF). Each member binds to different VEGF receptors (VEGFR): VGFR-A binds to VEGFR-1 and 2, while VEGF-B and PGF bind to VEGFR-1, and VEGF-C and D bind to VEGFR-2 and 3 [72].

Serum VEGF concentrations have been shown to be related to vascular involvement, metastasis and poor outcomes in patients with GC and GEJ cancers [73,74,75].

There are several anti-VEGF therapies which have been evaluated. These include the monoclonal antibody bevacizumab and multitarget tyrosine kinase inhibitors such as sunitinib and sorafenib.

### 4.1. Anti-VEGF mAb

#### 4.1.1. Bevacizumab (B)

B is a recombinant humanized monoclonal antibody that targets VEGF. Its combination with chemotherapy has shown to increase the anticancer activity in different tumours types [76,77,78].

A retrospective review of 16 patients who had received a combination of B combined with 5FU, leucovorin, and ox (FOLFOX-6) as first or advanced line for metastatic esophageal, GEJ cancer and GC showed interesting results. The DCR was 100% with 63% of PR and 37% of minor responses or SD. The median TTP and OS were 7 and 8.9 months respectively and in contrast to previous trials, there was no serious B-related toxicities [79].

There are several phase II and a phase III studies evaluating the efficacy of first-line B in patients with advanced GC and GEJ tumours. Globally when combined with chemotherapy as first-line the RR varies between 24–67% with TTP of 6.6–8.3 months and OS 11.1–12.3 months. 

Shah *et al*. carried out a multicenter phase II study with iri, cis, and B. After the evaluation of 47 patients, the RR was 65% and the median OS was 12.3 months (95% CI, 11.3 to 17.2 months). There was no increase in chemotherapy-related toxicity but B-related toxicity included a 28% incidence of grade 3 hypertension, two patients with gastric perforation (in the context of both disease progression and response to the treatment) and a single patient with a myocardial infarction. Grade 3 to 4 thromboembolic events occurred in 25% of patients. Of note is that though the primary tumor was unresected in 40 patients, only two patients showed significant upper gastrointestinal bleeding, one of them in the setting of anticoagulation therapy due to a pulmonary embolism [78].

Another phase II study with ox, docetaxel and B was performed in 38 patients. Though they observed DCR of 79% with median PFS of 6.6 months and OS of 11.1 months, two patients had gastrointestinal perforation. This fact raised safety-related concerns with B in first-line although no toxic deaths were recorded and its activity was promising [80].

When B was combined with docetaxel, cis and iri in GEJ cancers, the DCR was 93%. In this study all the patients were also treated with aspirin 81 mg daily unless clinically contraindicated, patients. The combination was globally well tolerated with 9% grade 4 thromboembolic events, but interestingly none of these in those patients taking aspirin [81].

The phase II study of modified DCF (docetaxel, cis, 5FU) with B in 42 patients with metastatic GEJ cancer showed a DCR of 89%, a 6-month PFS of 83%, and the median OS was not reached at the time of reporting. The OS rate was 75% at 12 months and 22% at 18 months. In this trial, side effects included asymptomatic venous thromboembolism (29%), and 1 patient had grade 3 upper gastrointestinal bleeding [82].

A further phase II trial with the same combination in 44 patients reported a RR of 67% with a median PFS of 12 months and OS of 16.2 months with 37% of OS at 2-year [83].

This promising activity was tested in a phase III clinical trial evaluating the combination of cape and cis with B or placebo as first line treatment for patients with advanced GC (AVAGAST). A total of 773 patients were enrolled. Significant superiority of B over placebo was not demonstrated. The median OS was 10.1 months with placebo and 12.1 months with B (hazard ratio 0.87; *p* = 0.1002) though the latter was associated with a significantly longer PFS (29.5 *vs*. 38.0 months; *p* = 0.0121) (See Table 5). The incidence of grade 3–4 toxicity was 0.5% in the placebo group and 6.2% in the B group. However, the incidence of arterial/venous thrombotic events and gastrointestinal perforation were, respectively, 15.2 and 2.1% in the placebo group *vs*. 9.6 and 1.3% in the B arm [84,85]. Regional differences were noted in a post hoc subgroup analysis. Asian-Pacific patients appeared to have better outcomes as measured by OS, independent of other prognostic variables. Patients from Europe/Americas with one or more unfavorable prognostic factors also appeared to derive a survival benefit from B (6.8 *vs.* 11.5 months in America and 12.1 *vs.* 13.9 in Europe) [85,86].

**Table 5:** Clinical trials with anti-VEGF agents for advanced GC and GEJ cancers.

Author	Type of study	N	Line	Type of chemo	% RR	TTP mos.	OS mos.
Shah *et al*. [78]	Phase II	47	First-line	Cis + iri + B	65	8.3	12.3
El Reyes *et al*. [81]	Phase II	38	First-line	Ox + docetaxel + B	59	6.6	11.1
Enzinger *et al*. [92]	Phase II	26	First-line	Docetaxel + cis + iri + B	24	_	_
AVAGAST [82,84,85,86,87]	Phase III	387	First-line	Cis + cape + B	46	6.7	12.1
		387		Cis + cape + placebo	37.4	5.3	10.1
Shah *et al*. [83]	Phase II	44	First-line	Docetaxel + cis + 5FU + B	67	12	16.2
Kim *et al*. [93]	Phase I	21	First-line	Sorafenib + capecit + cis	63	10	14.7
Sun *et al*. [94]	Phase II	44	First-line	Sorafenib + docetaxel + cis	39	5.8	13.6
Moehler *et al*. [96]	Phase II	38	Second-line	Sunitinib	5	1.5	6.3
Bang *et al*. [97]	Phase II	42	Second-line	Sunitinib	5	4.3	12.7
RAINBOW [90]	Phase III	_	Second-line	R + cis + fluoropyrimidine	_	_	_
Ongoing	Phase III	_	Third-line	Apatinib	_	_	_
				Placebo			

A report of AVAGAST biomarker analysis showed that a high plasma VEGF-A levels and a low tumour neuropilin (NRP-1 which is a co-receptor for VEGF-A) expression were associated with favorable outcomes and these biomarkers were most common in distal and diffuse tumours. These biomarkers therefore could provide a rationale for GC and/or subtype-specific outcomes with B [87].

Currently the ST03 study is ongoing. This is a multicentre, open-label, phase II/III randomised clinical trial aiming at assessing the safety and feasibility (in the stage I with the first 200 pts) and efficacy (stage II) of the addition of B to perioperative ECX chemotherapy. A translational study is also ongoing. The primary outcome for stage I (safety results including cardiac EF) have already been reported [88]. The rate of complications was similar for both arms with the most relevant with perforations at the primary tumor, cardiovascular events such as heart failure and arrhythmia, wound healing complications and gastrointestinal bleeding. 

The stage II primary outcome measure is OS and the secondary end-points are RR, resection rate, disease free survival, toxicity, and quality of life. The accrual is expected to be completed in 2013. An embedded pilot study within ST03 randomising HER2 positive patients to ECX ± L is planned. 

#### 4.1.2. Ramucirumab (R)

R is a fully human, IgG1 monoclonal antibody that inhibits VEGFR-2. Phase I clinical trials demonstrated its safety and efficacy in patients with advanced cancer refractory to standard chemotherapy [89]. The RAINBOW study, a randomized, multicenter, double-blind, placebo-controlled phase III clinical trial of weekly paclitaxel with or without R (IMC-1121B) is ongoing in patients with metastatic GC refractory to or progressive after first-line therapy with platinum and fluoropyrimidine [90,91]. Another phase III study is currently ongoing, although the recruiting of patients has been completed, to receive R or BSC [92].

### 4.2. Anti-VEGF TKI

#### 4.2.1. Sorafenib

Sorafenib is an oral multitargeted TKI that inhibits VEGFR-1, VEGFR-2, VEGFR-3, platelet derived growth factor (PDGFR), B-Raf, Raf-1 and c-Kit. In tumor xenograft models it is able to inhibit effectively tumor growth and angiogenesis. When it was combined with cape and cis for advanced GC in a first line phase I study, the RR was encouraging (62.5%) with a PFS of 10 months and OS of 14.7 months [93]. Sorafenib has been also evaluated in phase II clinical trials. Sun *et al*. carried out a study of sorafenib combined with 3-weekly docetaxel and cis. The results were interesting as they achieved an OS of 13.6 months but with a PFS of only 5.8 months, which is less than the PFS reported in a phase III study of chemotherapy alone. The authors suggested that this result could be due to the use of second-line chemotherapy [94].

Another phase II study carried out by a Spanish group was presented in ASCO annual meeting 2012. The study included 40 patients in second-line (36 were evaluable for response). The results showed a 47.2% of SD with one CR. The median PFS was 3 months and OS was 6.5 months. But in those cases in which PFS to first line was >6 months, OS of 9.7 months was achieved while only 5.6 months if PFS <6 months after first line therapy (*p* = 0.04). The authors concluded that the combination of ox and sorafenib in advanced GC patients previously treated with cis and fluoropyrimidine showed a safe profile and suggest that PFS under a cis-fluoropyrimidine-based first line therapy determine subgroups of GC patients with different clinical behaviors [95].

#### 4.2.2. Sunitinib

Sunitinib is another oral multitargeted TKI which targets RET, VEGFR-1, VEGFR-2, VEGFR-3, PDGFRα, PDGFRβ, Flt 3, c-KIT, and colony-stimulating factor receptor 1. This drug has been tested as monotherapy for second-line treatment of advanced GC showing modest activity. In a phase II trial conducted on 52 patients with chemo-refractory advanced GC, sunitinib monotherapy resulted in a median OS of 5.8 months. In a subgroup analyses, tumor VEGF-C expression compared with no expression was associated with significantly shorter median PFS (1.2 *vs*. 2.8 months, *p* = 0.0119) even if it was no difference in RR [96].

Another phase II study performed in 78 patients as second-line treatment showed minimal radiological RR (2.6%), with a PFS of 2.3 and OS of 6.8 months [97]. Although the toxicities were manageable, at this time, there is no plan to move forward with sunitinib in further clinical investigations of GC [98].

#### 4.2.3. Cediranib

Cediranib (AZD2171) is a highly potent inhibitor of VEGFR-1 and VEGFR-2 and it shows also activity against c-Kit and PDGFR-β [99]. This drug has been tested in a phase I study in combination with cis plus a fluoropyrimidine (S-1 or cape). 14 patients with advanced GC in first line were included and the tolerability was good being the most common side effects a decreased appetite, fatigue and nausea (92.9%) but the preliminary efficacy evaluation showed only one confirmed and three unconfirmed PR. Therefore additional studies with this drug are still expected [100]. 

#### 4.2.4. Apatinib

Apatinib is a TKI that selectively targets VEGFR-2. A randomized, three-arm phase II trial has evaluated apatinib as a third-line treatment for patients with advanced GC. 141 patients were recruited and received apatinib (850 mg. qd.), apatinib (425 mg. bid.), or placebo. The results showed the highest RR for the group with the lowest dose of apatinib (13%), with a DCR of 39.1%. The median PFS was similar for both groups with apatinib (3.4 months) and the median OS was 2.5, 4.8, and 4.3 months respectively for the three groups. Adverse reactions with this drug included mainly hypertension and hand-foot syndrome [101]. With these results a phase III clinical trial comparing apatinib to placebo also in a third-line setting in advanced GC is currently being conducted. The enrollment target is 270 and the patients are randomized to apatinib 850 mg qd oral or placebo [102].

#### 4.2.5. Telatinib

Telatinib is a highly selective, potent and orally available inhibitor of VEGFR, PDGFR and KIT tyrosine kinases. Telatinib, because of its highly selective nature appears well tolerated at high, continuous doses and exhibits no overlapping toxicities with chemotherapy. A phase II trial in combination with standard-of-care chemotherapy in first-line GC patients has been performed among 39 evaluable patients. 64% showed a PR and one patient had a CR. The overall DCR was of 92%. The median PFS was 140 days and the combination was well tolerated at the full recommended dose of telatinib. The most common side effects, such as hypertension and fatigue, were manageable and reversible [103]. Given these promising results, a phase III multicenter, double-blind, randomized trial evaluating telatinib in combination with cis and cape is planned. 

## 5. Other Targeted Agents

### 5.1. PI3k-Akt-mTOR-Targeted Therapy

#### Everolimus

Everolimus (RAD001) is an oral inhibitor of the mammalian target of rapamycin serine-threonine kinase (mTOR). This drug has shown to inhibit the PI3K/Akt/mTOR pathway, a key regulator of cell proliferation, metabolism, and angiogenesis, and has shown efficacy against GC in preclinical and phase I/II studies.

Yamada *et al*. carried out a phase II study to evaluate the activity of everolimus in 53 patients with previously treated metastatic GC. The results were presented in ASCO Gastrointestinal Cancers symposium 2009 and showed no objective responses but DCR of 55% with a median PFS of 83 days (95% CI 50–91 days) [104]. Takiuchi *et al*. demonstrated good activity with everolimus in previously treated advanced GC patients with SD of 56%, PFS 2.7 months, OS 10.1 months [105]. Both of these studies showed a good tolerance with stomatitis, anorexia, fatigue, rash and peripheral edema as the main adverse events. Only stomatitis and hyponatremia were the two main grade 3/4 side effects.

A phase III study (GRANITE-1) of 656 patients with advanced GC previously treated with one or two lines of systemic treatment has also been presented. No significant improvement in OS when patients received everolimus compared with BSC (5.4 *vs.* 4.3 months, respectively *p* = 0.1244). Everolimus did, however, reduce the risk of progression by 34% and the PFS was 1.7 *vs.* 1.4 months respectively (*p* = 0.0001). These results were consistent across the different subgroups of patients included and the everolimus safety profile was consistent with that previously seen in other trials [106] (See Table 6). Given the observed improvement in PFS, everolimus may be interesting in combination with other biological or chemotherapy agents in future studies, especially if evaluated in first-line therapy. 

**Table 6:** Clinical trials with everolimus in previously treated patients with GC.

Author	Type of study	Line	N	Type of chemo	% RR	TTP months	OS months
Doi *et al*. [104]	Phase II	Advanced	53	Everolimus	0	2.7	10.1
Van Cutsem *et al*. [106]	Phase III	Advanced	656	Everolimus+BSC	_	1.7	5.4 *
				BSC		1.4	4.3

* Non-statistically significant.

### 5.2. Polo-like Kinase 1 Inhibitors

Polo-like kinase 1 (Plk1) belongs to a family of serine/threonine kinases which have similar but non-overlapping functions in the cell cycle progression. Plk1 plays a key role to ensure the normal mitosis and is mainly expressed in proliferating tissues but overexpressed in cancers.

Overexpression of Plk1 could be also a prognostic marker for many cancers. In the study performed by Kanaji *et al*. in patients with GC, authors found that the prognosis of patients with PLK1-positive tumors was significantly worse than that of patients with PLK1-negative tumors (*p* < 0.05). Moreover, multivariate analysis demonstrated that PLK1 expression was an independent prognostic factor [107]. Several Plk1 inhibitors have been developed and tested for the cancer treatment. In a phase I study of dose escalation of the Plk1 inhibitor BI 2536, the maximum tolerated dose was defined at 100 mg for day 1 and 8 schedule. Patients were treated for up to eight courses without evidence of accumulating toxicity. This drug showed to have a favorable PK and safety profile at the tested dose and schedule [108].

### 5.3. HGF-c-Met Pathway and FGFR Pathway

c-Met, a cell surface receptor for hepatocyte growth factor (HGF), regulates multiple cellular processes such as cell proliferation, invasion and angiogenesis. Studies carried out *in vitro* and *in vivo* have shown its overexpression and activation in GC [109]. Autophosphorylation of c-met, a transmembrane tyrosine kinase, activates several signaling transduction cascades leading to cancer metastasis-cell proliferation, motility, invasion and angiogenesis.

To elucidate the role of c-met activation in GC invasion and liver metastasis, the functional expression and activation of c-met in GC cell lines and tumor tissues was evaluated. Authors found that c-met activation was strongly associated with GC invasion and liver metastasis [110]. Disappointingly, GSK1363089 a TKI of c-Met has shown no clinical benefits in patients with previously treated gastric cancer [111].

#### 5.3.1. Tivantinib

Tivantinib is a selective, non-ATP competitive, small-molecule inhibitor of c-MET. Elevated expressions of c-MET and its ligand, hepatocyte growth factor (HGF), have been frequently found in GC, and are associated with a more aggressive phenotype.

A single arm phase II study evaluating the efficacy of tivantinib as monotherapy in patients with metastatic GC previously treated evaluated thirty patients. No objective responses were obtained. The DCR was 36.7% with a median PFS of 43 days (95% CI: 29.0–92.0). This study carried out a pretreatment tumor tissue collection to evaluate the relationship between biomarkers and efficacy of this drug. c-MET gene amplification (defined as ≥5 copies/cell) was observed in 13.3%. Authors did not identify any relationship of treatment outcome with biomarkers including c-MET gene amplification, c-MET, p-c-MET and HGF expression in tumor.

Regarding toxicity, grade 3 or 4 adverse events occurred in 43.3% patients, being neutropenia and anemia recognized as drug-related, but fortunately there was no treatment-related death and novel safety concern was not recognized [112].

#### 5.3.2. Rilotumumab

The effectiveness of this agent, a mAb against HGF, was reported in a randomised phase II trial presented in ESMO congress 2011. The results were in favour of the combination with chemotherapy (ECX) compared with chemotherapy alone and more remarkable for c-Met overexpression patients established by IHC. Currently further randomised clinical trials targeting these population with overexpression of c-Met are being planned [113].

### 5.4. Fibroblast Growth Factor Receptor (FGFR)

#### Dovitinib

Dovitinib is a FGFR inhibitor which has shown clinical benefits in GC. It is being evaluated in a single-center, single-arm, open-label phase II trial as salvage treatment monotherapy for patients with metastatic or unresectable GC harboring FGFR2 amplification after failure of first or second line chemotherapy [114]. The results are still pending.

## 6. Conclusions and Future Directions

Despite all the efforts put in improving GC prognosis, it is still an aggressive disease with a high mortality rate. Moreover most of the patients will present with an advanced disease at diagnosis which makes even more pessimistic the situation. Even though, improvements in GC therapy are expected as our comprehension of GC molecular biology and signaling pathways ameliorates [115]. If in the past the combination of several chemotherapeutic agents prolonged the OS, at present we look at the integration of targeted therapies which has shown promising results over the past decade [116].

In fact, the addition of trastuzumab to combination chemotherapy is now considered a standard first-line treatment for HER2 positive advanced GC patients and this has led to a new era in the treatment of this disease. Moreover this treatment is still under investigation for potential use in perioperative settings.

Lapatinib is currently one of the most promising new agents for this disease with two phase III clinical trials ongoing in selected patients with HER-2 positivity. The LOGIC trial in first-line setting with capecitabine and cisplatin and the TYTAN in second-line with paclitaxel.

As opposed to this the agents targeting human EGFR remain very controversial in treating GC. While cetuximab combined with standard chemotherapy has shown promising results in phase II trial, the phase III EXPAND failed in prolonging the PFS and OS when compared to chemotherapy alone. Whether this negative result could be due to the lack of patients selection based on the presence of reliable predictive biomarkers is something that deserves further attention and research. In addition, the REAL-III trial, another phase III evaluating the benefit of adding panitumumab to a combination of chemotherapy not only failed in showing any advantage but also showed a worsening of OS and PFS. Authors concluded that these findings may be in part due to lowered doses of chemotherapy used in this regimen but the reality is that further studies are still necessary to draw a definitive conclusion. 

More promising, however, seems to be the combination with chemotherapy and matuzumab but it has only been evaluated in phase II trials.

Antiangiogenic therapy has shown only marginal effectiveness over existing treatments for GC despite all the efforts done, and the main cause for these negative results has been as well the lack of predictive biomarkers for these drugs activity which would allow better selection of the targeted population to be treated. Although biomarkers such as serum VEGF-A and microvessel density have repeatedly been reported as potentially useful predictive markers for the effectiveness of these anti-angiogenic treatments, they remain still unconfirmed by phase III trials which makes it really difficult to establish which agents might be most beneficial to certain patients.

The majority of existing targeting agents focus on both EGF/VEGF and their receptors, but more recent research has revealed many new pathways related to tumor angiogenesis and proliferation, providing numerous new potential targets. In this way, several trials are ongoing to test potential targeting agents addressed to the downstream components of VEGFR/EGFR, such as inhibitors of mTOR, c-Met, and HDAC. The phase III trial of everolimus (GRANITE-1) has reported prolonged PFS at ASCO Gastrointestinal Cancers Symposium this year with a reduction of 34% of the risk of progression. What is relevant is the point that generally the treatments are well tolerated with no unexpected toxicities and most of them easy manageable.

Finally, drug resistance is still a critical issue in the development of molecular targeted agents. In fact, effective treatment may well involve a combination of different targeted agents, or targeted agents with several regimens of chemotherapies, or it may require the use of sequential molecular treatments as part of complex approaches to cancer therapy.

Globally, it remains really clear that further studies are necessary to optimize the usage of anticancer drugs in clinical settings but the development of reliable biomarkers become a key point to better selection of a tailored therapy for individual patients which may also significantly improve treatment safety and patient survival.

## Appendix

Summary of all the clinical trials sorted by molecular agent and year of publication.

**Table A1:** Summary of clinical trials.

Author/Study/Ref.	Type of study	Year	Molecular agent
Shah *et al.* [78]	Phase II	2006	Cetuximab
Pinto *et al*. FOLCETUX [20]	Phase II	2007	Cetuximab
Wöll *et al*. [27]	Phase II	2008	Cetuximab
Zhang *et al*. [26]	Phase II	2008	Cetuximab
Pinto *et al*. DOCETUX [21]	Phase II	2009	Cetuximab
Han *et al*. [22]	Phase II	2009	Cetuximab
Kanzler *et al*. [24]	Phase II	2009	Cetuximab
Yeh *et al*. [25]	Phase II	2009	Cetuximab
Enzinger *et al*. [30]	Phase II	2010	Cetuximab
Kim *et al*. [23]	Phase II	2011	Cetuximab
MerckSerono.EXPAND [31]	Phase III	2012	Cetuximab
Okines *et al*. REAL-III [40-42]	Phase II-III	2010	Panitumumab
Rao *et al*. MATRIX [47]	Phase II	2010	Matuzumab
Sun *et al*. [94]	Phase II	2010	Sorafenib
Bang *et al*. ToGA [8]	Phase III	2010	Trastuzumab
Enzinger *et al*. [81]	Phase II	2008	Bevacizumab
El Rayes *et al*. [80]	Phase II	2010	Bevacizumab
AVAGAST [84,85,86,87]	Phase III	2011	Bevacizumab
Shah *et al*. [78]	Phase II	2011	Bevacizumab
Iqbal *et al*. [64]	Phase II	2007	Lapatinib
Lenz *et al*. [67]	Phase II	2010	Lapatinib
TYTAN [69]	Phase III	2010	Lapatinib
Galsky *et al*. [65]	Phase II	2012	Lapatinib
LOGIC [70]	Phase III	ongoing	Lapatinib
Kim *et al*. [43]	Phase II	2011	Nimotuzumab
Wang *et al*. [44]	Phase II	2012	Nimotuzumab
Moehler *et al.* [96]	Phase II	2011	Sunitinib
Bang *et al*. [97]	Phase II	2011	Sunitinib
Doi *et al*. [104]	Phase II	2010	Everolimus
Van Cutsem *et al*. [106]	Phase III	2012	Everolimus
RAINBOW [90]	Phase III	2012	Ramucirumab

## References

[1] Ferlay J., Shin H.R., Bray F., Forman D., Mathers C., Parkin D.M. (2010). Estimates of worldwide burden of cancer in 2008: GLOBOCAN 2008. Int. J. Cancer.

[2] Arak A., Kull K. (1994). Factors influencing survival of patients after radical surgery for gastric cancer. Ann. Oncol..

[3] Cunningham D., Allum W.H., Stenning S.P., Thompson J.N., van de Velde C.J., Nicolson M., Scarffe J.H., Lofts F.J., Falk S.J., Iveson T.J. (2006). Perioperative chemotherapy *versus* surgery alone for respectable gastroesophageal cancer. N. Engl. J. Med..

[4] Sugano K. (2008). Gastric cancer: Pathogenesis, screening and treatment. Gastrointest. Endosc. Clin. N. Am..

[5] Macdonald J.S., Smalley S.R., Benedetti J., Hundahl S.A., Estes N.C., Stemmermann G.N., Haller D.G., Ajani J.A., Gunderson L.L., Jessup J.M. (2001). Chemoradiotherapy after surgery compared with surgery alone for adenocarcinoma of the stomach or gastroesophageal junction. N. Engl. J. Med..

[6] Cunningham D., Starling N., Rao S., Iveson T., Nicolson M., Coxon F., Middleton G., Daniel F., Oates J., Norman A.R. (2008). Capecitabine and Oxaliplatin for Advanced Esophagogastric Cancer. N. Engl. J. Med..

[7] Wagner A.D., Grothe W., Haerting J., Kleber G., Grothey A., Fleig W.E. (2006). Chemotherapy in advanced gastric cancer: A systematic review and meta-analysis based on aggregate data. J. Clin. Oncol..

[8] Bang Y.J., van Cutsem E., Feyereislova A., Chung H.C., Shen L., Sawaki A., Lordick F., Ohtsu A., Omuro Y., Satoh T. (2010). T in combination with chemotherapy *versus* chemotherapy alone for treatment of HER2-positive advanced gastric or gastro-oesophageal junction cancer (ToGA): A phase 3, open-label, randomised controlled trial. Lancet.

[9] Carpenter G., Cohen S. (1990). Epidermal growth factor. J. Biol. Chem..

[10] Herbst R.S. (2004). Review of epidermal growth factor receptor biology. Int. J. Radiat. Oncol. Biol. Phys..

[11] Oda K., Matsuoka Y., Funahashi A., Kitano H. (2005). A comprehensive pathway map of epidermal growth factor receptor signaling. Mol. Syst. Biol..

[12] Barnham K.J., Torres A.M., Alewood D., Alewood P.F., Domagala T., Nice E.C., Norton R.S. (1998). Role of the 6-20 disulfide bridge in the structure and activity of epidermal growth factor. Protein Sci..

[13] Rojo F., Albanell J., Sauleda S., Baselga J. (2001). Characterization of epidermal growth factor (EGF) receptor and transforming growth factor (TGF) alpha expression in gastric cancer and its association with activation of mitogen-activated protein kinase (MAPK). Proc. Am. Soc. Clin. Oncol..

[14] Gamboa-Dominguez A., Dominguez-Fonseca C., Quintanilla-Martinez L., Reyes-Gutierrez E., Green D., Angeles-Angeles A., Busch R., Hermannstädter C., Nährig J., Becker K.F. (2004). Epidermal growth factor receptor expression correlates with poor survival in gastric adenocarcinoma from Mexican patients: A multivariate analysis using a standardized immunohistochemical detection system. Mod. Pathol..

[15] Wang K.L., Wu T.T., Choi I.S., Wang H., Resetkova E., Correa A.M., Hofstetter W.L., Swisher S.G., Ajani J.A., Rashid A. (2007). Expression of epidermal growth factor receptor in esophageal and esophagogastric junction adenocarcinomas: Association with poor outcome. Cancer.

[16] Kim J.W., Kim H.P., Im S.A., Kang S., Hur H.S., Yoon Y.K., Oh D.Y., Kim J.H., Lee D.S., Kim T.Y. (2008). The growth inhibitory effect of lapatinib, a dual inhibitor of EGFR and HER2 tyrosine kinase, in gastric cancer cell lines. Cancer Lett..

[17] Galizia G., Lieto E., Orditura M., Castellano P., Mura A.L., Imperatore V., Pinto M., Zamboli A., de Vita F., Ferraraccio F. (2007). Epidermal growth factor receptor (EGFR) expression is associated with a worse prognosis in gastric cancer patients undergoing curative surgery. World J. Surg..

[18] Lieto E., Ferraraccio F., Orditura M., Castellano P., Mura A.L., Pinto M., Zamboli A., de Vita F., Galizia G. (2008). Expression of vascular endothelial growth factor (VEGF) and epidermal growth factor receptor (EGFR) is an independent prognostic indicator of worse outcome in gastric cancer patients. Ann. Surg. Oncol..

[19] Martinelli E., de Palma R., Orditura M., de Vita F., Ciardiello F. (2009). Anti-epidermal growth factor receptor monoclonal antibodies in cancer therapy. Clin. Exp. Immunol..

[20] Pinto C., di Fabio F., Siena S., Cascinu S., Rojas Llimpe F.L., Ceccarelli C., Mutri V., Giannetta L., Giaquinta S., Funaioli C. (2007). Phase II study of cetuximab in combination with FOLFIRI in patients with untreated advanced gastric or gastroesophageal junction adenocarcinoma (FOLCETUX study). Ann. Oncol..

[21] Pinto C., di Fabio F., Barone C., Siena S., Falcone A., Cascinu S., Rojas-Llimpe F.L., Stella G., Schinzari G., Artale S. (2009). Phase II of cetuximab in combination with cis/docetaxel as first-line treatment in patients with unresectable/metastatic gastric or gastroesophageal junction (GEJ) adenocarcinoma (DOCETUX study). Br. J. Cancer.

[22] Han S.W., Oh D.Y., Im S.A., Park S.R., Lee K.W., Song H.S., Lee N.S., Lee K.H., Choi I.S., Lee M.H. (2009). Phase II study and biomarker analysis of cetuximab in combination with modified FOLFOX6 in advanced gastric cancer. Br. J. Cancer.

[23] Kim C., Lee J.L., Ryu M.H., Chang H.M., Kim T.W., Lim H.Y., Kang H.J., Park Y.S., Ryoo B.Y., Kang Y.K. (2011). A prospective phase II study of cetuximab in combination with XELOX (capecitabine and oxaliplatin) in patients with metastatic and/or recurrent advanced gastric cancer. Invest. New Drugs.

[24] Kanzler S., Trarbach T., Seufferlein T., Kubicka S., Lordick F., Geissler M., Daum S., Galle P.R., Moehler M. (2009). Cetuximab with irinotecan/folinic acid/5-FU as first line treatment in advanced gastric cancer: A nonrandomized multicenter AIO phase II study. J. Clin. Oncol..

[25] Yeh K., Hsu C., Hsu C., Lin C., Shen Y., Wu S., Chiou T., Chao Y., Cheng A. (2009). Phase II study of cetuximab plus weekly cis and 24-h infusion of high-dose 5-fluorouracil and leucovorin for the first-line treatment of advanced gastric cancer. J. Clin. Oncol..

[26] Zhang X., Xu J., Shen L., Wang J., Liang J., Xu N., Bai Y., Wang J. (2008). A phase II study of cetuximab with cis and capecitabine as first-line treatment in advanced gastric cancer. J. Clin. Oncol..

[27] Wöll E., Greil R., Eisterer W., Fridrik M., Grünberger B., Gattringer K., Mayrbäurl B., Russ G., Thaler J. (2008). Oxaliplatin, iri, and cetuximab in advanced gastric cancer. First efficacy results of a multicenter phase II trial of the AGMT. J. Clin. Oncol..

[28] Okines A., Cunningham D., Chau I. (2011). Targeting the human EGFR family in esophagogastric cancer. Nat. Rev. Clin. Oncol..

[29] Cappetta A., Lonardi S., Pastorelli D., Bergamo F., Lombardi G., Zagonel V. (2012). Advanced gastric cancer (GC) and cancer of the gastro-oesophageal junction (GEJ): Focus on targeted therapies. Crit. Rev. Oncol. Hematol..

[30] Enzinger P.C., Burtness B., Hollis D., Niedzwiecki D., Ilson D., Benson A.B., Mayer R.J., Goldberg M. (2010). CALGB 80403/ECOG 1206: A randomized phase II study of three standard chemotherapy regimens (ECF, IC, FOLFOX) plus cetuximab in metastatic esophageal and GE junction cancer. J. Clin. Oncol..

[31] Leach B. Cetuximab Fails to Extend PFS in Advanced Gastric Cancer. http://www.onclive.com/web-exclusives/Cetuximab-Fails-to-Extend-PFS-in-Advanced-Gastric-Cancer/.

[32] Safran H., Suntharalingam M., Dipetrillo T., Ng T., Doyle L.A., Krasna M., Plette A., Evans D., Wanebo H., Akerman P. (2008). Cetuximab with concurrent chemoradiation for esophagogastric cancer: Assessment of toxicity. Int. J. Radiat. Oncol. Biol. Phys..

[33] Chan J.A., Blaszkowsky L.S., Enzinger P.C., Ryan D.P., Abrams T.A., Zhu A.X., Temel J.S., Schrag D., Bhargava P., Meyerhardt J.A. (2011). A multicenter phase II trial of single-agent cetuximab in advanced esophageal and gastric adenocarcinoma. Ann. Oncol..

[34] Gold P.J., Goldman B., Iqbal S., Leichman L.P., Zhang W., Lenz H.J., Blanke C.D. (2010). Cetuximab as second-line therapy in patients with metastatic esophageal adenocarcinoma: A phase II Southwest Oncology Group Study (S0415). J. Thorac. Oncol..

[35] Tebbutt N.C., Sourjina T., Strickland A.H., van Hazel G.A., Pavlakis N., Ganju V., Murone C., MacGregor D., Gebski V., Cummins M. (2008). ATTAX2: Docetaxel plus cetuximab as second-line treatment for docetaxel-refractory oesophago-gastric cancer-final results of a multicentre phase II trial by the AGITG. J. Clin. Oncol..

[36] Stein A., Al-Batran S.E., Arnold D., Peinert S., Siewczynski R., Schmoll H.J. (2007). Cetuximab with irinotecan as salvage therapy in heavily pretreated patients with metastatic gastric cancer. J. Clin. Oncol..

[37] Lin L., Hecht J. (2000). A phase III trial of iri in patients with advanced adenocarcinoma of the gastroesophageal junction. J. Clin. Oncol..

[38] Thuss-Patience P.C., Kretzschmar A., Deist T., Hinke A., Bichev D., Lebedinzew B., Schumacher G., Gebauer B., Maier V., Reichardt P. (2009). Irinotecan *versus* best supportive care (BSC) as second-line therapy in gastric cancer: A randomized phase III study of the Arbeitsgemeinschaft Internistische Onkologie (AIO). Proc. Am. Soc. Clin. Oncol..

[39] Sartore-Bianchi A., Ricotta R., Cerea G., Maugeri M.R., Siena S. (2007). Rationale and clinical results of multi-target treatments in oncology. Int. J. Biol. Markers..

[40] Okines A.F., Ashley S.E., Cunningham D., Oates J., Turner A., Webb J., Saffery C., Chua Y.J., Chau I. (2010). Epirubicin, oxaliplatin, and capecitabine with or without panitumumab for advanced esophagogastric cancer: Dose-finding study for the prospective multicenter, randomized, Phase II/III REAL-3 Trial. J. Clin. Oncol..

[41] Chau I., Okines A.F.C., González de Castro D., Saffery C., Barbachano Y., Wotherspoon L., Puckey L., Hulkki Wilson S., Coxon F.Y., Middleton G.W. (2011). REAL3: A multicenter randomized phase II/III trial of epirubicin, oxaliplatin, and capecitabine (EOC) *versus* modified (m) EOC plus panitumumab (P) in advanced oesophagogastric (OG) cancer. Response rate (RR), toxicity, and molecular analysis from phase II. J. Clin. Oncol..

[42] Waddell T.S., Chau I., Barbachano Y., González de Castro D., Wotherspoon A., Saffery C, Middleton G.W., Wadsley J., Ferry D.R., Mansoor W. (2012). A randomized multicenter trial of epirubicin, oxaliplatin, and capecitabine (EOC) plus panitumumab in advanced esophagogastric cancer (REAL3). J. Clin. Oncol..

[43] Kim Y.H., Sasaki Y., Lee K.H., Rha S.Y., Park S., Boku N., Komatsu Y., Kim T., Kim S., Sakata Y. (2011). Randomized phase II study of nimotuzumab, an anti-EGFR antibody, plus iri in patients with 5-fluorouracil-based regimen-refractory advanced or recurrent gastric cancer in Korea and Japan: Preliminary results. J. Clin. Oncol..

[44] Wang J.W., Chi Y., Zheng Z., Qu T., Zhou A., Yang L., Jiang W., Shi S., Sun Y., Song Y., Kang S. (2012). Randomized, single-centered, phase II clinical trial of nimotuzumab plus cis and S-1 as first-line therapy in patients with advanced gastric cancer. J. Clin. Oncol..

[45] Rao S., Starling N., Cunningham D., Benson M., Wotherspoon A., Lüpfert C., Kurek R., Oates J., Baselga J., Hill A. (2008). Phase I study of epirubicin, cis and capecitabin plus matuzumab in previously untreated patients with advanced oesophagogastric cancer. Br. J. Cancer.

[46] Cunningham D., Starling N., Rao S., Iveson T., Nicolson M., Coxon F., Middleton G., Daniel F., Oates J., Norman A.R. (2008). Capecitabine and oxaliplatin for advanced esophagogastric cancer. N. Engl. J. Med..

[47] Rao S., Starling N., Cunningham D., Sumpter K., Gilligan D., Ruhstaller T., Valladares-Ayerbes M., Wilke H., Archer C., Kurek R. (2010). Matuzumab plus epirubicin, cis and capecitabine (ECX) compared with epirubicin, cis and capecitabine alone as first-line treatment in patients with advanced oesophago-gastric cancer: A randomised, multicentre open-label phase II study. Ann. Oncol..

[48] Herbst R.S., Fukuoka M., Baselga J. (2004). Anovel targeted approach to treating cancer. Nat. Rev. Cancer.

[49] Rojo F., Tabernero J., Albanell J., van Cutsem E., Ohtsu A., Doi T., Koizumi W., Shirao K., Takiuchi H., Ramon y Cajal S. (2006). Pharmacodynamic studies of gefitinib in tumor biopsy specimens from patients with advanced gastric carcinoma. J. Clin. Oncol..

[50] Rodriguez C.P., Adelstein D.J., Rice T.W., Rybicki L.A., Videtic G.M., Saxton J.P., Murthy S.C., Mason D.P., Ives D.I. (2010). A phase II study of peri-operative concurrent chemotherapy, gefitinib, and hyperfractionated radiation followed by maintenance gefitinib in locoregionally advanced esophagus and gastroesophageal junction cancer. J. Thorac. Oncol..

[51] Dragovich T., McCoy S., Fenoglio-Preiser C.M., Wang J., Benedetti J.K., Baker A.F., Hackett C.B., Urba S.G., Zaner K.S., Blanke C.D. (2006). Phase II trial of erlotinib in gastroesophageal junction and gastric adenocarcinomas: SWOG 0127. J. Clin. Oncol..

[52] Wainberg Z.A., Lin L.S., DiCarlo B., Dao K.M., Patel L., Park D.J., Wang H.J., Elashoff R., Ryba N., Hecht J.R. (2010). Final results of a phase II study of modified FOLFOX6 and erlotinib in patients with metastatic adenocarcinoma of the esophagus and gastroesophageal junction. J. Clin. Oncol..

[53] Meza-Junco M., Sawyer M.B. (2012). Metastatic gastric cancer-focus on targeted therapies. Biologics.

[54] Cidon E.U., Centeno R.G., Lagarto E.G., Peral J.I. (2011). HER-2 evaluation in a specific gastric cancer population with the highest rate of mortality in Spain. J. Oncol..

[55] Fornaro L., Lucchesi M., Caparello C., Vasile E., Caponi S., Ginocchi L., Masi G., Falcone A. (2011). Anti-HER agents in gastric cancer: From bench to bedside. Nat. Rev. Gastroenterol. Hepatol..

[56] Meza-Junco J., Au H.J., Sawyer M.B. (2011). Critical appraisal of T in treatment of advanced stomach cancer. Cancer Manag. Res..

[57] Park D.I., Yun J.W., Park J.H., Oh S.J., Kim H.J., Cho Y.K., Sohn C.I., Jeon W.K., Kim B.I., Yoo C.H. (2006). HER-2/neu amplification is an independent prognostic factor in gastric cancer. Dig. Dis. Sci..

[58] Lordick F., Bang Y.J., Kang Y.K., Otero Reyes D., Manikhas G.M., Shen L. (2007). HER2-positive advanced gastric cancer: Similar HER2-positivity levels to breast cancer. Eur. J. Cancer.

[59] Kang Y., Bang Y., Lordick F., Park S., Sawaki A., Chung H., Shen L., Xu J.M., Leon-Chong J., van Cutsem E. (2008). Incidence of gastric and gastroesophageal cancer in the ToGa trial: Correlation with HER2 positivity. Gastrointestinal Cancers Symposium (ASCO).

[60] Kim S.Y., Kim H.P., Kim Y.J., Oh do Y., Im S.A., Lee D., Jong H.S., Kim T.Y., Bang Y.J. (2008). T inhibits the growth of human gastric cancer cell lines with HER2 amplification synergistically with cis. Int. J. Oncol..

[61] Fujimoto-Ouchi K., Sekiguchi F., Yasuno H., Moriya Y., Mori K., Tanaka Y. (2007). Antitumor activity of T in combination with chemotherapy in human gastric cancer xenograft models. Cancer Chemother. Pharmacol..

[62] Cortés-Funes H., Rivera F., Alés I., Márquez A., Velasco A., Colomer R., García-Carbonero R., Sastre J., Guerra J., Grávalos C. (2007). Phase II of T and cis in patients (pts) with advanced gastric cancer (AGC) with HER2/neu overexpression/amplification. J. Clin. Oncol..

[63] Safran H., Dipetrillo T., Akerman P., Ng T., Evans D., Steinhoff M., Benton D., Purviance J., Goldstein L., Tantravahi U. (2007). Phase I/II study of T, paclitaxel, cis and radiation for locally advanced, HER2 overexpressing, esophageal adenocarcinoma. Int. J. Radiat. Oncol. Biol. Phys..

[64] Iqbal S., Goldman B., Lenz H.J., Fenoglio-Preiser C.M., Blanke C.D. (2007). S0413: A phase II SWOG study of GW572016 (lapatinib) as first line therapy in patients (pts) with advanced or metastatic gastric cancer. J. Clin. Oncol..

[65] Galsky M.D., von Hoff D.D., Neubauer M., Anderson T., Fleming M., Nagarwala Y., Mahoney J.M., Midwinter D., Vocila L., Zaks T.Z. (2012). Target-specific, histology independent, randomized discontinuation study of lapatinib in patients with HER2-amplified solid tumors. Invest. New Drugs.

[66] Hecht J.R., Urba S.G., Koehler M., Ellis C., Gagnon R., Kemner A., Versola N., Spector M., Press M.F., Richel D.J. (2008). Lapatinib monotherapy in recurrent upper gastrointestinal malignancy: Phase II efficacy and biomarker analyses. Gastrointestinal Cancers Symposium (ASCO).

[67] Lenz H.J., Zhang W., Kemner A.M., Kaneko T., Yang D., Franklin N., Moon H.  (2010). Lapatinib 1 capecitabine in advanced gastric cancer: An open-label phase II study of non ERBB2-targeted disease. European Society of Medical Oncology Congress (ESMO).

[68] Roth A., Moehler M.H., Mauer M., Schad A., Karrasch M., Praet M., Lim M.L., Das-Gupta A., Lutz M.P. (2010). Lapatinib in combination with ECF/X in EGFR1 or HER2-overexpressing first-line metastatic gastric cancer (GC): A phase II randomized placebo controlled trial (EORTC 40071). J. Clin. Oncol..

[69] Satoh T., Bang Y., Wang J., Xu J., Chung H.C., Yeh K., Chen J., Mukaiyama A., Yoshida P., Ohtsu A. (2010). Interim safety analysis from TYTAN: A phase III Asian study of lapatinib in combination with paclitaxel as second-line therapy in gastric cancer. J. Clin. Oncol..

[70] LOGiC-Lapatinib Optimization Study in ErbB2 (HER2) Positive Gastric Cancer: A Phase III Global, Blinded Study Designed to Evaluate Clinical Endpoints and Safety of Chemotherapy Plus Lapatinib. http://clinicaltrials.gov/ct2/show/NCT00680901?term=LOGiC&rank=8/.

[71] Ferrara N., Davis-Smyth T. (1997). The biology of vascular endothelial growth factor. Endocr. Rev..

[72] Yoshikawa T., Tsuburaya A., Kobayashi O., Sairenji M., Motohashi H., Yanoma S., Noguchi Y. (2000). Plasma concentration of VEGF and bFGF in patients with gastric carcinoma. Cancer Lett..

[73] Maeda K., Chung Y.S., Ogawa Y., Takatsuka S., Kang S.M., Ogawa M., Sawada T., Sowa M. (1996). Prognostic value of vascular endothelial growth factor expression in gastric carcinoma. Cancer.

[74] Karayiannakis A.J., Syrigos K.N., Polychronidis A., Zbar A., Kouraklis G., Simopoulos C., Karatzas G. (2002). Circulating VEGF levels in the serum of gastric cancer patients: Correlation with pathological variables, patient survival, and tumor surgery. Ann. Surg..

[75] Hurwitz H., Fehrenbacher L., Novotny W., Cartwright T., Hainsworth J., Heim W., Berlin J., Baron A., Griffing S., Holmgren E. (2004). Bevacizumab plus iri, fluorouracil, and leucovorin for metastatic colorectal cancer. N. Engl. J. Med..

[76] Sandler A., Gray R., Perry M.C., Brahmer J., Schiller J.H., Dowlati A., Lilenbaum R., Johnson D.H., Sandler A., Gray R. (2006). Paclitaxel-carboplatin alone or with bevacizumab for non-small-cell lung cancer. N. Engl. J. Med..

[77] Miller K., Wang M., Gralow J., Dickler M., Cobleigh M., Perez E.A., Shenkier T., Cella D., Davidson N.E. (2007). Paclitaxel plus bevacizumab *versus* paclitaxel alone for metastatic breast cancer. N. Engl. J. Med..

[78] Shah M.A., Ramanathan R.K., Ilson D.H., Levnor A., D’Adamo D., O’Reilly E., Tse A., Trocola R., Schwartz L., Capanu M. (2006). Multicenter phase II study of iri, cis, and bevacizumab in patients with metastatic gastric or gastroesophageal junction adenocarcinoma. J. Clin. Oncol..

[79] Cohenuram M.K., Lacy J. (2008). FOLFOX6 and bevacizumab (FOLFOX6/B) for metastatic esophageal, gastroesophageal (GE) and gastric cancer (G) adenocarcinoma: A single institution’s initial clinical experience. Gastrointestinal Cancers Symposium (ASCO).

[80] El-Rayes B.F., Zalupski M., Bekai-Saab T., Heilbrun L.K., Hammad N., Patel B., Urba S., Shields A.F., Vaishampayan U., Dawson S. (2010). A phase II study of bevacizumab, oxaliplatin, and docetaxel in locally advanced and metastatic gastric and gastroesophageal junction cancers. Ann. Oncol..

[81] Enzinger P.C., Ryan D.P., Regan E.M., Lehman N., Abrams T.A., Hezel A.F., Fidias P., Sequist L.V., Blaszkowsky L.S., Fuchs C.S. (2008). Phase II trial of docetaxel, cis, iri, and bevacizumab in metastatic esophagogastric cancer. J. Clin. Oncol..

[82] Ohtsu A., Shah M.A., van Cutsem E., Rha S.Y., Sawaki A., Park S.R., Lim H.Y., Yamada Y., Wu J., Langer B. (2011). Bevacizumab in combination with chemotherapy as first-line therapy in advanced gastric cancer: A randomized, double-blind, placebo-controlled phase III study. J. Clin. Oncol..

[83] Shah M.A., Jhawer M., Ilson D.H., Lefkowitz R.A., Robinson E., Capanu M., Kelsen D.P. (2011). Phase II study of modified docetaxel, cis, and fluorouracil with bevacizumab in patients with metastatic gastroesophageal adenocarcinoma. J. Clin. Oncol..

[84] Shah M.A., van Cutsem E., Kang Y.K., Dakhil S.R., Satoh T., Chin K., Bang Y.J., Bu L., Bilic G., Ohtsu A. (2012). Survival analysis according to disease subtype in AVAGAST: First-line capecitabine and cis plus bevacizumab or placebo in patients with advanced gastric cancer. Gastrointestinal Cancers Symposium (ASCO).

[85] Kang Y., Ohtsu A., van Cutsem E., Rha S.Y., Sawaki A., Park S., Lim H., Wu J., Langer B., Shah M.A. (2010). AVAGAST: A randomized, double-blind, placebo-controlled, phase III study of first-line capecitabine and cis plus bevacizumab or placebo in patients with advanced gastric cancer (AGC). J. Clin. Oncol..

[86] Kang Y., Ohtsu A., van Cutsem E., Roman L., Nunes J., Li C., Otero D., Rivera F., Aprile G., Pimentel Alvarez P.R. (2011). Survival analysis by pooling risk factors in AVAGAST: First-line capecitabine plus bevacizumab or placebo in patients with advanced gastric cancer. J. Clin. Oncol..

[87] Shah M., Kang Y.K., Ohtsu A., Delmar P., Foernzler D., Langer B., Scherer S., van Cutsem E. (2010). Tumor and blood plasma biomarker analyses in the AVAGAST phase III randomized study of first-line bevacizumab + capecitabine/cis in patients with advanced gastric cancer. European Society of Medical Oncology Congress (ESMO).

[88] Smyth E.C., Langley R.E., Stenning S.P., Stevenson L., Allum W.H., Grabsch H., Alderson D., Riddell A.M., Crosby T., Mason R. (2012). ST03: A randomized trial of perioperative epirubicin, cis plus capecitabine (ECX) with or without bevacizumab (B) in patients (pts) with operable gastric, oesophagogastric junction (OGJ) or lower oesophageal adenocarcinoma. J. Clin. Oncol..

[89] Spratlin J.L., Cohen R.B., Eadens M., Gore L., Camidge D.R., Diab S., Leong S., O’Bryant C., Chow L.Q., Serkova N.J. (2010). Phase I pharmacologic and biologic study of ramucirumab (IMC-1121B), a fully human immunoglobulin G1 monoclonal antibody targeting the vascular endothelial growth factor receptor-2. J. Clin. Oncol..

[90] A Study of Paclitaxel With or Without Ramucirumab in Metastatic Gastric adenocarcinoma (RAINBOW). http://clinicaltrials.gov/show/NCT01170663/.

[91] Wilke H., Cunningham D., Ohtsu A., Nuber U., Bruns R., Schmitt-Bormann B. (2012). A randomized, multicenter, double-blind, placebo (PBO)-controlled phase III study of paclitaxel (PTX) with or without ramucirumab (IMC-1121B; RAM) in patients (pts) with metastatic gastric adenocarcinoma, refractory to or progressive after first-line therapy with platinum (PLT) and fluoropyrimidine (FP). J. Clin. Oncol..

[92] Study of IMC-1121B (Ramucirumab) With Best Supportive Care in Patients With Gastric Cancer and Adenocarcinoma. http://clinicaltrials.gov/show/NCT00917384/.

[93] Kim C., Lee J.L., Choi Y.H., Kang B.W., Ryu M.H., Chang H.M., Kim T.W., Kang Y.K. (2012). Phase I dose-finding study of sorafenib in combination with capecitabine and cis as a first-line treatment in patients with advanced gastric cancer. Invest. New Drugs..

[94] Sun W., Powell M., O’Dwyer P.J., Catalano P., Ansari R.H., Benson A.B. (2010). Phase II study of sorafenib in combination with docetaxel and cis in the treatment of metastatic or advanced gastric and gastroesophageal junction adenocarcinoma: ECOG 5203. J. Clin. Oncol..

[95] Gallego R., Martin-Richard M., Pericay C., Garcia-Foncillas J., Queralt B., Casado E., Feliu J., Vega I., Juez I., Visa L. (2012). Phase II study of oxaliplatin and sorafenib in advanced gastric cancer after failure of cis-fluoropyrimidine-based (PF) treatment. J. Clin. Oncol..

[96] Moehler M., Mueller A., Hartmann J.T., Ebert M.P., Al-Batran S.E., Reimer P., Weihrauch M., Lordick F., Trarbach T., Biesterfeld S. (2011). An open-label, multicentre biomarker-oriented AIO phase II trial of sunitinib for patients with chemo-refractory advanced gastric cancer. Eur. J. Cancer.

[97] Bang Y.J., Kang Y.K., Kang W.K., Boku N., Chung H.C., Chen J.S., Doi T., Sun Y., Shen L., Qin S. (2011). Phase II study of sunitinib as second-line treatment for advanced gastric cancer. Invest. New Drugs.

[98] Liu L., Wu N., Li J. (2012). Novel targeted agents for gastric cancer. J. Hematol. Oncol..

[99] Lindsay C.R., MacPherson I.R., Cassidy J. (2009). Current status of cediranib: The rapid development of a novel anti-angiogenic therapy. Future Oncol..

[100] Satoh T., Yamada Y., Muro K., Hayashi H., Shimada Y., Takahari D., Taku K., Nakajima T.E., Shi X., Brown K.H. (2011). Phase I study of cediranib in combination with cis plus fluoropyrimidine (S-1 or capecitabine) in Japanese patients with previously untreated advanced gastric cancer. Cancer Chemother. Pharmacol..

[101] Tian S., Quan H., Xie C., Guo H., Lü F., Xu Y., Li J., Lou L. (2011). YN968D1 is a novel and selective inhibitor of vascular endothelial growth factor receptor-2 tyrosine kinase with potent activity *in vitro* and *in vivo*. Cancer Sci..

[102] Phase III Study of Apatinib Tablets in the Treatment of Advanced or Metastatic Gastric Cancer. http://clinicaltrials.gov/ct2/show/NCT01512745/.

[103] TEL0805 trial: A phase 2 study of telatinib (TEL) in combination with capecitabine (X) and cis (P) as first-line treatment in patients (pts) with advanced gastric or gastro-esophageal junction (GEJ) cancer. A Study of Telatinib in Combination with Chemotherapy in Subjects With Advanced Gastric Cancer. http://clinicaltrials.gov/show/NCT00952497/.

[104] Doi T., Muro K., Boku N., Yamada Y., Nishina T., Takiuchi H., Komatsu Y., Hamamoto Y., Ohno N., Fujita Y. (2010). Multicenter phase II study of everolimus in patients with previously treated metastatic gastric cancer. J. Clin. Oncol..

[105] Takiuchi H., Doi T., Muro K., Yamada Y., Boku N., Nishina T., Komatsu Y., Hamamoto Y., Ohno N., Ohtsu A. (2010). Everolimus in patients with previously treated metastatic gastric cancer: Final results of a multicenter phase II study. Gastrointestinal Cancers Symposium (ASCO).

[106] Van Cutsem E., Yeh K.H., Bang Y.J., Shen L., Ajani J.A., Bai Y., Chung H.C., Pan H.M., Chin K., Muro K. (2012). Phase III trial of everolimus (EVE) in previously treated patients with advanced gastric cancer (AGC): GRANITE-1. J. Clin. Oncol..

[107] Kanaji S., Saito H., Tsujitani S., Matsumoto S., Tatebe S., Kondo A., Ozaki M., Ito H., Ikeguchi M. (2006). Expression of Polo-Like Kinase 1 (PLK1) Protein Predicts the Survival of Patients with Gastric Carcinoma. Oncology.

[108] Hofheinz R., Hochhaus A., Al-Batran S., Nanci A., Reichardt V., Trommeshauser D., Hoffmann M., Steegmaier M., Munzert G., Jäger E. (2006). A phase I repeated dose escalation study of the Polo-like kinase 1 inhibitor BI 2536 in patients with advanced solid tumours. J. Clin. Oncol..

[109] Sierra J.R., Tsao M.S. (2011). c-MET as a potential therapeutic target and biomarker in cancer. Ther. Adv. Med. Oncol..

[110] Cho H., Zhang X., Jung M., Kwon W., Jung J., Jeung H., Roh J.H., Chung H.C. (2010). C-met as a therapeutic target for metastatic potential of gastric cancer. J. Clin. Oncol..

[111] Jhawer M., Kindler H.L., Wainberg Z., Ford J., Kunz P., Tang L., McCallum S., Kallender H., Shah M.A. (2009). Assessment of two dosing schedules of GSK1363089 (GSK089), a dual MET/VEGFR2 inhibitor, in metastatic gastric cancer (GC): Interim results of a multicenter phase II study. J. Clin. Oncol..

[112] Muro K., Ryu M.H., Yasui H., Nishina T., Ryoo B.Y., Boku N., Kang Y.K. (2012). A phase II study of tivantinib monotherapy in patients with previously treated advanced or recurrent gastric cancer. J. Clin. Oncol..

[113] Iveson T., Donehower R.C., Davidenko I., Tjulandin S., Deptala A., Harrison M., Loh E., Jiang Y., Oliner K., Dubey S. (2011). Safety and Efficacy of Epirubicin, Cis, and Capecitabine (ECX) Plus Rilotumumab (R) as First-line Treatment for Unresectable Locally Advanced (LA) or Metastatic (M) Gastric or Esophagogastric Junction (EGJ) Adenocarcinoma. European Society of Medical Oncology Congress (ESMO).

[114] Kang Y.K. A Phase II Trial of Dovitinib Monotherapy as Salvage Treatment in Patients With Metastatic or Unresectable Gastric Cancer Harboring FGFR2(Fibroblst Growth Factor Receptor 2) Amplification After Failure of First or Second Line Chemotherapy. http://clinicaltrials.gov/ct2/show/NCT01719549/.

[115] Zagouri F., Papadimitriou C.A., Dimopoulos M.A., Pectasides D. (2011). Molecularly targeted therapies in unresectable-metastatic gastric cancer. A systematic review. Cancer Treat. Rev..

[116] Benson A.B. (2008). Advanced Gastric Cancer: An Update and Future Directions. Gastrointest. Cancer Res..

